# Pixels of Possibility: AI Art and the Visual Reimagination of Successful Aging

**DOI:** 10.1093/geroni/igaf122.2251

**Published:** 2025-12-31

**Authors:** Jiayu Chen, Qingwei Wang

**Affiliations:** National University of Singapore, Singapore, Singapore; Xi’an Jiaotong-Liverpool University, Suzhou, Jiangsu, China (People’s Republic)

## Abstract

This article explores AI art’s cultural implications for reimagining successful aging. Despite AI’s increasing presence in artistic and everyday visual culture, its role in challenging dominant aging narratives and broadening representations of later life remains largely underexplored. This research takes an interdisciplinary approach, bridging gerontology, visual cultural studies, and AI research to investigate whether and how AI art can serve as a medium for shaping the narratives around successful aging. Through a qualitative case study of Auntieverse, an AI art project featuring elderly female figures, this research employs an innovative three-study design to examine the intersections of AI aesthetics, artistic intention, and audience reception. The findings reveal that while Auntieverse aims to disrupt ageist stereotypes by portraying older women with extreme autonomy, it also uncovers algorithmic biases in AI aesthetics and the interpretive gaps between artist and audience. There main themes emerged through iterative analysis of multimodal Data: “Re-seeing Age Identity,” which explores how AI-generated imagery reshapes representations of aging; “Re-thinking the Aging Body,” which examines AI aesthetics’ role in mediating perceptions of physical aging; and “‘Re-pAInting’ Successful Aging,” which considers AI art’s potential to either reinforce or redefine prevailing narratives of autonomy in later life. By positioning AI art as an active cultural force, this study demonstrates AI art’s potential role in contributing to gerontology through an in-depth case study. It advocates for a more critical and intentional engagement with AI’s influence on aging representation, through which the visual discourse surrounding successful aging could be challenged and reimagined.

